# Statistical optimization and gamma irradiation on cephalosporin C production by *Acremonium chrysogenum* W42-I

**DOI:** 10.1186/s13568-023-01645-5

**Published:** 2023-12-11

**Authors:** Asmaa A. Ibrahim, Ghadir S. El-Housseiny, Khaled M. Aboshanab, Ansgar Startmann, Mahmoud A. Yassien, Nadia A. Hassouna

**Affiliations:** 1 Department of Microbiology and Immunology, Faculty of Pharmacy, Ain Shams University, Organization of African Unity St., POB: 11566, Abbassia, Cairo Egypt; 2 W42 Industrial Biotechnology GmbH, 44227 Dortmund, Germany

**Keywords:** Cephalosporin C, *Acremonium chrysogenum*, Response surface methodology, Gamma, Irradiation

## Abstract

Most antibiotics now used in clinical practice are cephalosporins. *Acremonium (A.) chrysogenum* W42-I is an intermediate strain out of W42 strain improvement program whose productivity is above that of the wild-type strain to produce the broad-spectrum antibacterial cephalosporin C (CPC). As a result, fermentation process optimization is considered because it offers the ideal environment for strains to reach their full potential. Our research aimed to combine a rational design to regulate the fermentation process environment and culture media as well as to develop mutants with high productivity. Different media were tested to obtain maximum CPC production. To maximize the production of CPC, some environmental parameters were experimentally optimized via the Box-Behnken design used for response surface methodology (RSM). There were 17 tests conducted, and each experiment's reaction was recorded. Improvement of the CPC production was further achieved via mutagenesis using gamma radiation. Results revealed that a pH of 4, an incubation period of 4 days, and an inoculum size of 1% v/v using the optimized media (CPC2) were the optimum conditions for enhancing the CPC production by 4.43-fold. In addition, gamma irradiation further enhanced production to reach 3.46-fold using an optimum dose of 2 KGy. In conclusion, in comparison to initial production levels, CPC production increased 4.43-fold because of nutritional and environmental optimization. The mutant AC8 demonstrated a roughly 3.46-fold increase in activity against its parent type. Moreover, subsequent AC8 mutant culture demonstrated excellent genetic stability.

**Supplementary Information:**

The online version contains supplementary material available at 10.1186/s13568-023-01645-5.

## Keypoints

- Production of Cephalosporin C was statistically optimized using the Box-Behnken design.
- Optimum environmental conditions resulted in 4.43-fold increase in CPC production.
- Gamma irradiation further enhanced production to reach 3.46-fold using an optimum dose of 2 KGy.

**Supplementary Information:**

The online version contains supplementary material available at 10.1186/s13568-023-01645-5.

## Introduction

From a medical, economic, and academic perspective, β-lactams are the most significant antibiotics. They are the safest and most effective antibiotics that are currently available. Cephalosporins are low-toxic, broad-spectrum antibiotics that work similarly to ampicillin. Both Gram-positive and Gram-negative bacteria are sensitive to them (Adinarayana et al. 2003). The major component of all commercial semi-synthesized cephalosporins, 7-ACA (7-aminocephalosporanic acid), is derived from cephalosporin C (CPC). In submerged fermentations, industrial *A. chrysogenum* strains are the only source of the β-lactam antibiotic CPC (Barber et al. 2004). Despite some advantages, such as staphylococcal penicillinase resistance or selective toxicity, the CPC was used as a precursor for synthesizing semisynthetic cephalosporins due to its weak antibacterial efficacy (Williams and Geddes 1976).

Due to the economic impact and practicality of the method, optimizing fermentation conditions is crucial in any industrial production of natural active ingredients. Even though it is widely acknowledged that the one-dimensional examination is practically unable to attain an adequate optimum in a limited number of experiments, one-dimensional searches with consecutive variations in factors, such as the one factor at a time technique (OFAT), are still used. Single-factor optimization techniques are time-consuming and risk misinterpretation of results, particularly when they neglect the interactions between many components (He et al. 2004). For decades, statistical experimental designs have been used, and they can be applied at various stages of an optimization strategy, such as screening trials or looking for the best conditions for a specific response (Box and Behnken 1960). It is a progressive approach to optimize fermentation utilizing a statistically planned experiment (Ahsan et al. 2017; Ibrahim and Elkhidir 2010). This method not only assists the industry in developing the optimal media containing low-cost substrates but also utilizes the optimum environmental factors to maximize antibiotic production.

One of the useful techniques for increasing microbial production is still mutation. Random mutagenesis is thought to be a useful strategy for increasing the output of industrial strains (Parekh et al. 2000). Some product regulatory mutants were discovered to produce colonies with different morphologies, so morphologically changed mutant are greatly necessary in enhancing the strain activities (Adrio and Demain 2006).

The current study's primary goal was improvement of CPC production from *A. chrysogenum* W42-I through optimization of the nutritional and environmental parameters of the fermentation process and gamma irradiation for random mutagenesis of the producer organism.

## Materials and methods

### Microorganisms

*Acremonium (A.) chrysogenum* W42-I (W42 GmbH, strain collection) was kindly provided by Dr Ansgar Stratmann, a Founder and a General Manager (CEO) of the company W42 Industrial Biotechnology GmbH, 44,227 Dortmund, Germany. *A. chrysogenum* W42-I is a filamentous fungus that produces CPC. It is obtained from a rational strain development program–a result of a series of mutation/screening rounds of the *A. chrysogenum* wild strain, a CPC producer and its source is the ‘W42 strain collection (https://www.w42biotechnology.de/). In addition, this strain was deposited in the Culture Collection Ain Shams University (CCASU) of the World Data Centre for Microorganisms (WDCM) under the code, *Acremonium chrysogenum* isolate CCASU-2020-W42-I. (http://ccinfo.wdcm.org/collection/by_id/1186 (accessed on 25 Juni 2023).

It was stocked in tryptic soy broth (TSB) including 50% glycerol at a temperature of − 20 °C (Hopwood & Wright 1978; Kieser et al. 2000). *Staphylococcus aureus* ATCC 25923 was maintained in Luria–Bertani broth (LB broth) (Miller 1983) containing 50% glycerol at − 20 °C and for the bioassay of CPC production, by agar well diffusion technique, it was cultivated on nutrient agar at 37 °C for 24 h (Heatley 1944).

### Inoculum

*A. chrysogenum* W42-I spore suspension was obtained by vigorously shaking 3 day old culture slants for one minute with sterile saline solution (0.9% NaCl). Spores count was determined by haemocytometer (Liu 2016) and adjusted to roughly 10^8^ spores/mL. (Lotfy 2007).

### Culture media

The basal medium composition for *A. chrysogenum* W42-I was as follows (g/L): Dextrin, 35.0; Glucose, 7.5; (NH_4_)_2_SO_4_, 12.0; Urea, 1.2; L-methionine, 0.5; CaCO_3_, 6.0; (MgSO_4_)_7_ H_2_O, 4.8; KH_2_PO_4_, 0.6; K_2_HPO_4_, 1.2; corn steep liquor, 30.0; soybean meal, 24.0; dissolved in tap water and adjusted to pH 6.7. After sterilization for 20 min at 121 °C and cooling, sterile soybean oil, 1% v/v was added.

### Reverse phase HPLC analysis

High performance liquid chromatography (HPLC) was used to verify the production of CPC by the studied strain *A. chrysogenum* W42-I using Nucleosil 120 3C18, 3 µm, 125 mm × 4 mm column with pre-column, 10 mm × 4 mm, temperature 30 °C and 260 nm UV detector. The mobile phase was 2% acetonitrile in 20 mM ammonium acetate adjusted pH to 5.7 at 1.2 mL/min flow rate. Samples of 500 µL are cleared by centrifugation (10 min at 10,000*g*) and diluted 1:10 with mobile phase before analysis. Standard CPC (50 mg/L) are prepared in mobile phase of which 10 µL was injected, and duration time was 10 min. Additional filtration (0,45 µm) was recommended and the Standard CPC (1 g/L) was prepared in mobile phase and could be stored at − 20 °C.

### Determination of the antibacterial activity

The culture broth of *A. chrysogenum* W42-I and its mutants was centrifuged and filter-sterilized (0.22 µm pore size cellulose membrane filter, CHMLAB, Barcelona, Spain) to study the antibacterial activity of the CPC generated by these strains. Using the agar well diffusion technique (Heatley 1944), the culture filtrate was bio-assessed against *S. aureus* ATCC 25923. The Mueller Hinton agar surface (MHA, Difco, USA) was evenly and aseptically covered with a suspension of *S. aureus* ATCC 25923 (0.5 McFarland), and 200 µL of the culture filtrate was utilized to fill the wells. For at least 30 min, plates were kept between 4 and 8 degrees Celsius to allow culture filtrate to diffuse. After 24 h of incubation at 37 °C, the sizes of the inhibition zones were measured.

### Estimation of CPC

**Fig. 1:**
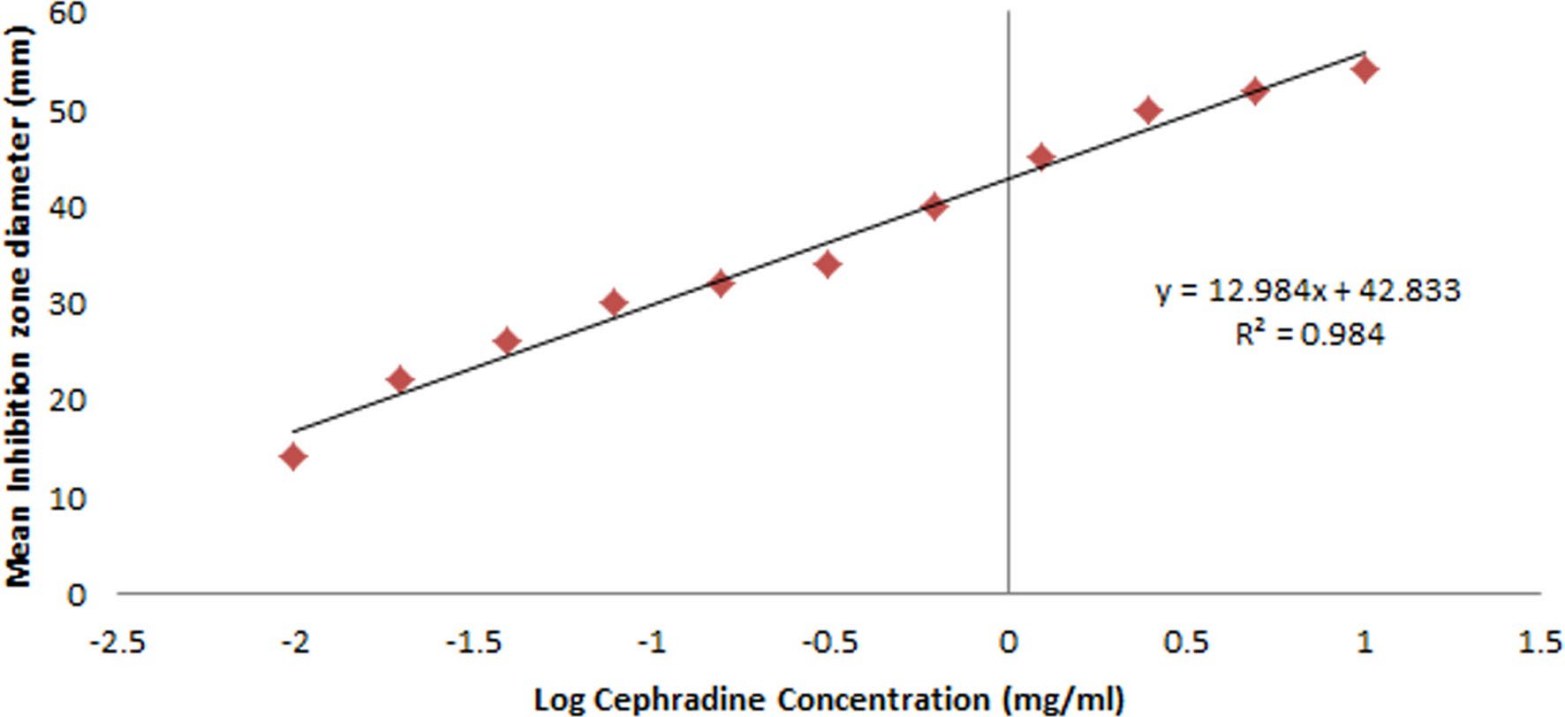
Standard Curve of standard cephradine antibacterial activity against *S. aureus* ATCC 25923

Since CPC was not commercially available, the standard cephradine (Sigma Aldrich, Cairo, Egypt) which has comparable antimicrobial activities to CPC was used. The cephradine concentrations were plotted against the corresponding inhibition zone diameters to create the standard curve. The proposed linear Eq. (1) was used to calculate CPC concentrations from this standard curve:

1 $$y = 12.984x + 42.833$$

y is the mean inhibition zone diameter (mm) with R^2^ = 0.984

x is log the cephradine concentration in mg/mL as shown in Fig. 1.

### Optimization of culture media

The seed culture was prepared by inoculating 2.5% v/v of *A.chrysogenum* W42-I spore suspension (10^8^ spores/mL) in 250 mL flasks containing 25 mL of basal medium with different L-methionine or soybean oil concentrations as listed in Additional file 1: Table S1 and incubating at 28 °C and 250 rpm. After 4 days of incubation, antibacterial activity was determined as mentioned above. The optimum media was selected for further experiments.

### Optimization of different environmental conditions

#### Response surface methodology (RSM)

The statistical software package Design Expert v. 7.0 (Stat-Ease Inc., Statistics Made Easy, Minneapolis, MN, USA) was used to optimize factors such as pH (coded variable A), inoculum size (coded variable B), and incubation period (coded variable C). The Box-Behnken design was employed to increase CPC productivity, with five replicates at the center point, resulting in 17 experiments (Additional file 1: Table S2). The average antibacterial activity of CPC represented by inhibition zone diameters was recorded as the dependent variable or response (Y).

#### Experimental confirmation test of RSM findings

The statistical numerical optimization function in the Design Expert software was conducted to generate a set of optimum culturing conditions, and the CPC produced in the shaking flask and compared to the un-optimized conditions.

#### Investigations of the runs using graphical and statistical analysis

All experiments were performed in triplicate; the values generated and presented are the means of the triplicate results, with error bars reflecting the standard deviation of the data.

All data investigations, response surfaces, and model diagnostic charts were created using Design Expert v. 7.0 (DesignExpert Software, Stat-Ease Inc., Statistics Made Easy, Minneapolis, MN, USA).

#### Mutation using gamma irradiation

*A. chrysogenum* W42-I was grown in 25 mL TSB for 5 days at a temperature of 28 °C and an agitation rate of 250 rpm (Clais et al. 2015). To find the optimum dose for mutation that achieved 99.99% death, five different gamma radiation doses (1, 2, 3, 4, and 5 KGy) were applied to five mL aliquots from this culture (10^8^ CFU/mL). At the time of the experiment, an Indian gamma cell producing 60Co radiation at a dosage rate of 2.2 KGy/h served as the source of gamma radiation. The National Center for Radiation Research and Technology, Atomic Energy Authority, Nasr City, Cairo, Egypt, performed the irradiation procedure. The irradiated cell culture was appropriately diluted after mutagenesis, plated on TSA plates, and incubated at 28 °C to produce single colonies. Tests were conducted on certain mutants for their enhanced CPC production.

## Results

### Reverse phase HPLC analysis

As shown in Additional file 1: Fig. S1, the HPLC analysis revealed the CPC peak of the *A. chrysogenum* W42-I at the same retention time as the standard CPC (Additional file 1: Fig. S1). The different peaks and their corresponding retention times and peak areas are displayed in Table 1.

**Table 1:** Different peaks and their and their corresponding retention times and peak areas eluted from the extract of *A. chrysogenum* W42-I

Peak name	Retention time (min)	Area (mV.s)	Area (%)
1	2.38	6.430	3.84
2. DAC	2.73	15.013	8.95
3	3.67	8.887	5.30
4	4.96	475	0.28
5	6.31	654	0.39
6. CPC	7.02	136.208	81.24

DAC, Deacetylcephalosporin; CPC, cephalosporin C

### Effect of different L-methionine and soybean oil concentrations on CPC production

**Fig. 2:**
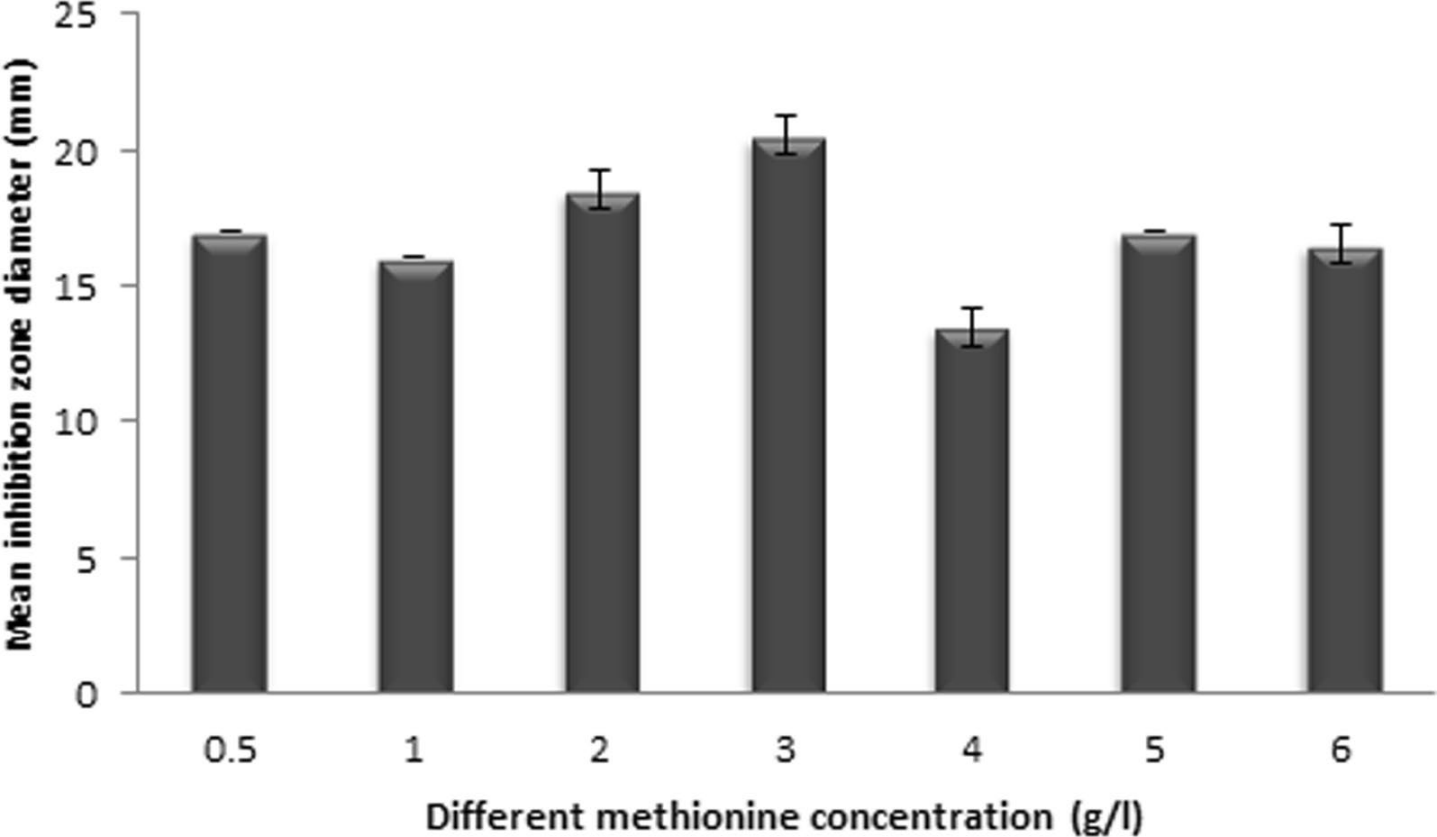
Effect of various L-methionine concentrations on CPC production

**Fig. 3:**
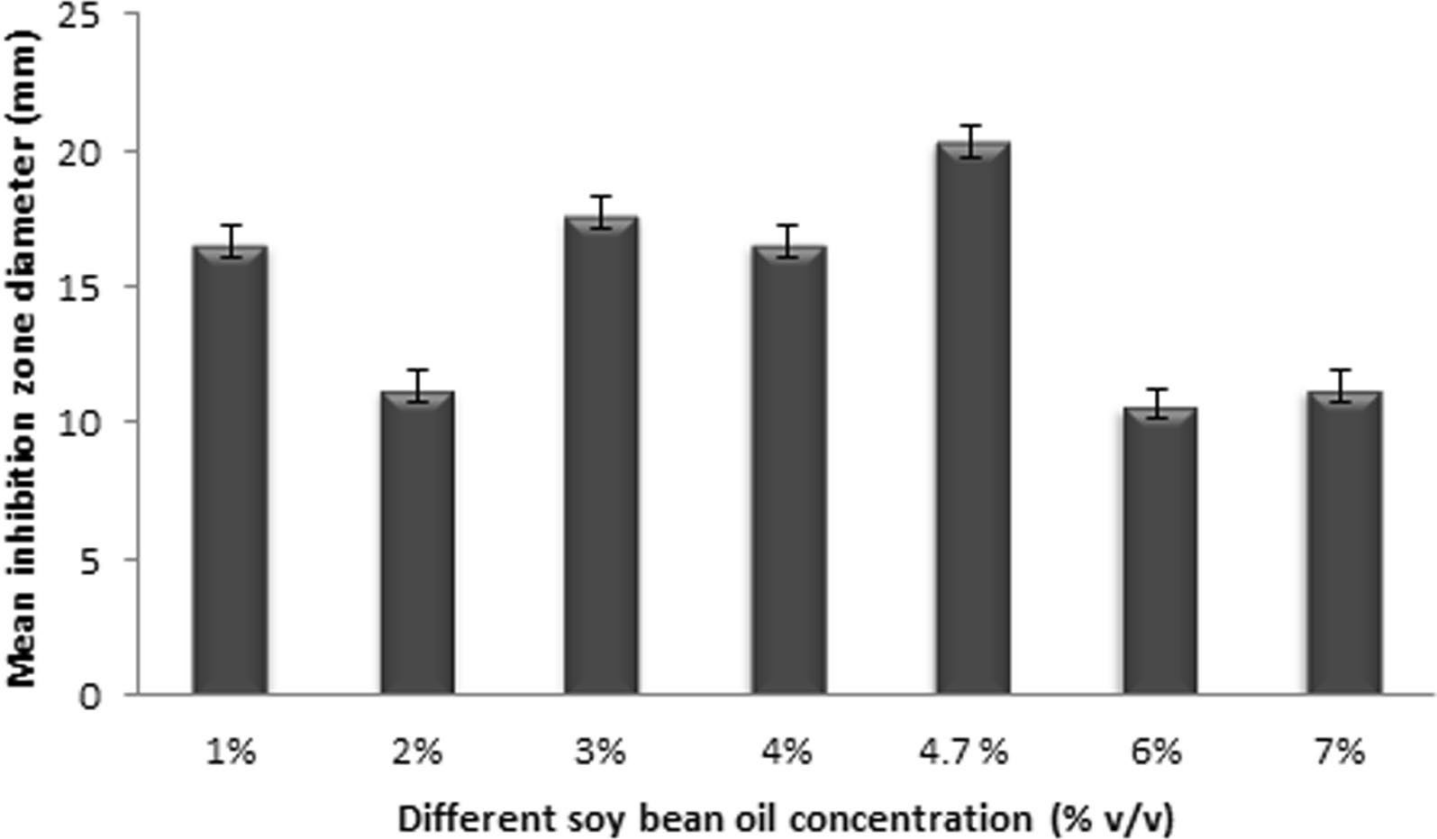
Effect of various soybean oil concentrations on CPC production

As shown in Figs. 2, 3, basal media resulted in a CPC activity equivalent to 9 µg/mL cephradine concentration. The highest CPC production was obtained at L-methionine and soybean oil concentrations of 3 g/L and 4.7% v/v, respectively, reaching 18.49 µg/mL cephradine concentration.

### Enhancement of CPC production using response surface methodology

The design, values and data obtained by the Design Expert software are presented in Table 2. Using the data gathered from the 17 experiments, the software identified the link between the factors and fitted an equation. The software generated the following RSM model equation [Eq. (2)] for CPC activity as a result.

**Table 2:** The observed and predicted responses of the Box Behnken design

Run order	pH	Inoculum size (% v/v)	Incubation period (days)	Observed response	Predicted response
1	6.5	1	1	0	2.50
2	9	2.5	1	16	15.63
3	6.5	2.5	3.5	18	19.44
4	6.5	2.5	3.5	19	19.44
5	9	2.5	6	0	0.88
6	6.5	2.5	3.5	20	19.44
7	4	2.5	6	20	19.13
8	4	2.5	1	16	13.87
9	6.5	4	1	0	0
10	6.5	1	6	20	21.25
11	9	1	3.5	17	14.82
12	6.5	2.5	3.5	20	19.44
13	6.5	2.5	3.5	19	19.44
14	4	1	3.5	24	23.07
15	9	4	3.5	16	15.82
16	6.5	4	6	0	1.25
17	4	4	3.5	22	24.07
$$\begin{aligned} {\text{CPC activity}}\; = & \; - \;{26}.{81167}\; + \;{1}.{15}000 \;*{\text{A}}\; + \;{11}.{3}0000\;*{\text{B}}\; + \;{2}0.00{111} \;*{\text{C}}\; \\ - & \;0.{8}0000\;*{\text{A}}\;*{\text{C}}\; - \;{3}.{13333}\;*{\text{B}}\;*{\text{ C}}\; - \;{1}.{13111}\;*{\text{C2}} \\ \end{aligned}$$ (2)					

Additional file 1: Table S3 displays the ANOVA results. ANOVA validates the models and explains the relevance of the factors influencing CPC production (El Housseiny et al. 2016). The P value was used to determine the relevance of each of the coefficients. The Model F rating of 48.33 for CPC production indicated that the model is noteworthy. There is only a 0.01% possibility of a ''Model F value'' this large occurring owing to noise (P value < 0.0001). Furthermore, A, C, AC, BC, and C2 were discovered to be significant model terms in this situation (Additional file 1: Table S3). A low coefficient of variation (CV) of 11.55% was obtained, indicating that the experimental values were reliable. The model fit was given with a coefficient of determination R2 of 0.9699, suggesting that the model could explain 96.99% of the variability in the response. The predicted R-squared (Pred. R2) value of 0.7533 was determined to be in reasonable agreement with the adjusted R-squared (Adj. R2) value of 0.9498. An adequate precision ratio of 21.475 was obtained in this model, indicating an appropriate signal. This model is useful for navigating the design space.

**Fig. 4:**
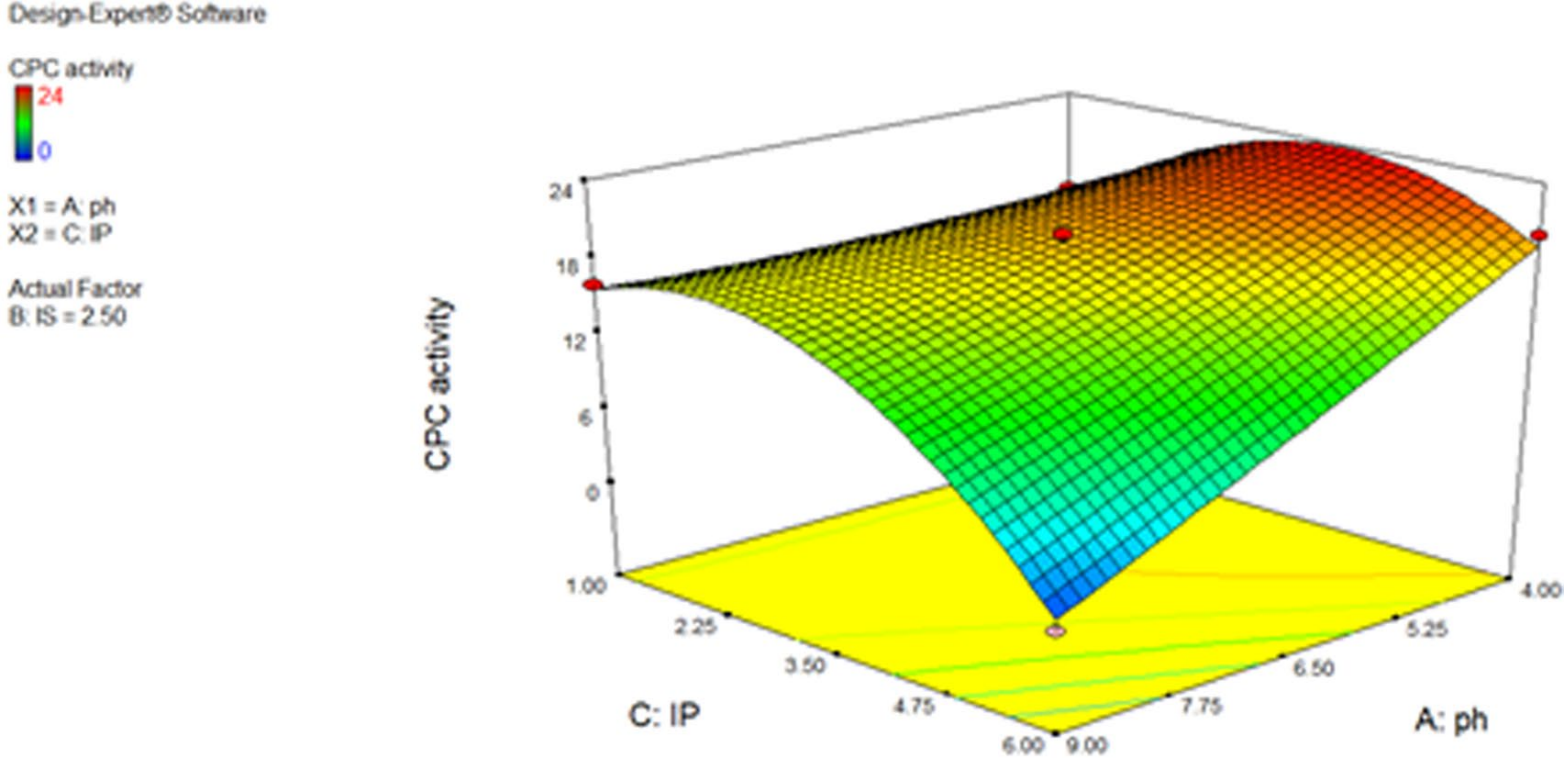
Three-dimensional (3D) surface for the effects of inoculum size, incubation period and pH on CPC activity. The remaining parameter (Inoculum size IS) was adjusted to central level when the influence of two parameters (Incubation period IP and pH) was plotted

**Fig. 5:**
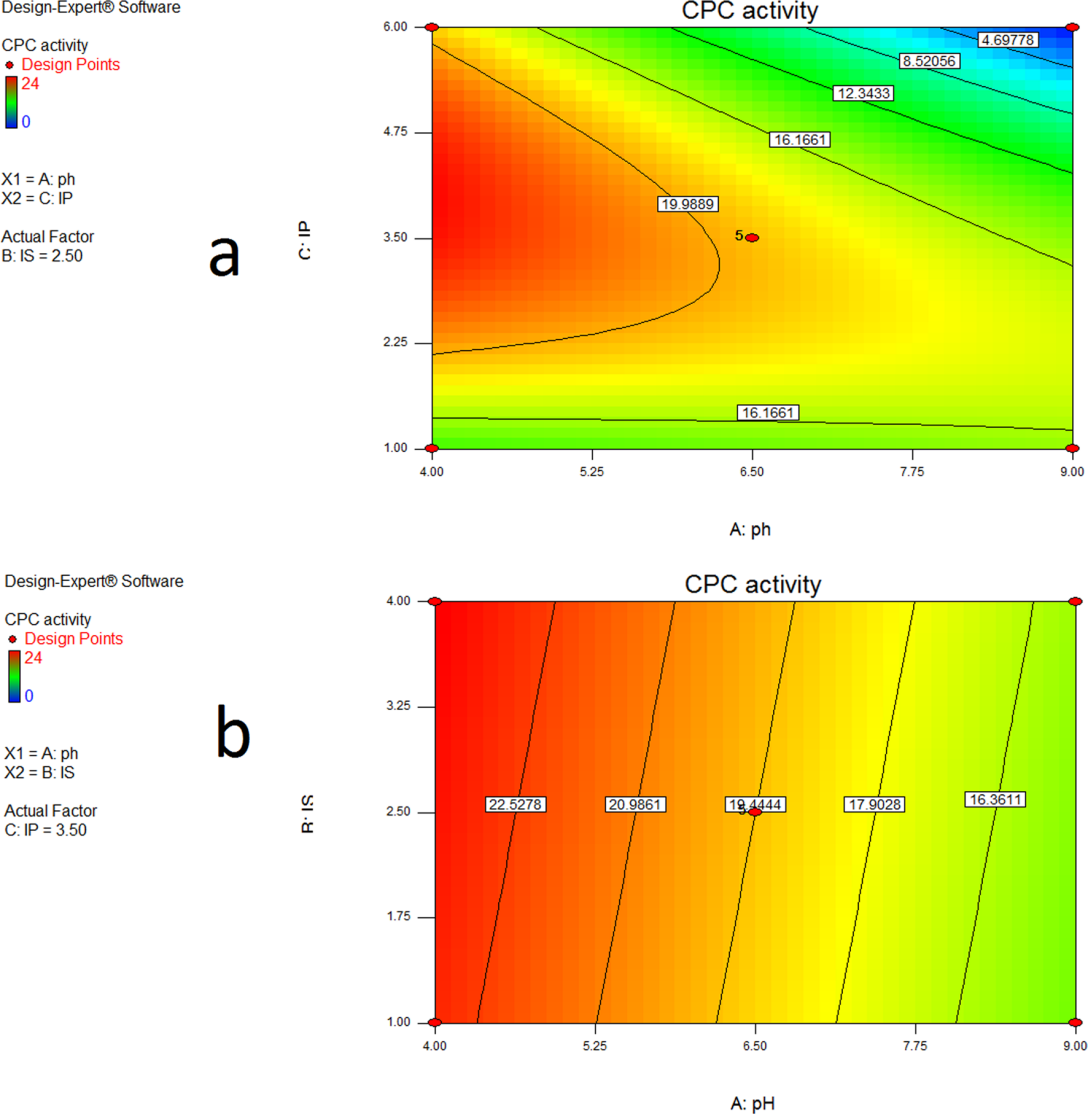
**a**, **b** Contour plots for the effects of inoculum size, incubation period and pH on CPC activity. When two parameters' effects were plotted, the other ones were set to central level

From the three-dimensional (3D) and contour plots between the input factors (Figs. 4, 5), as well as by using the numerical optimization tool in the Design Expert software, the ideal conditions for maximum CPC production were determined to be a pH of 4, an inoculum size of 1%v/v, and an incubation period of 4 days.

### Model diagnostics

The following graphical summaries for case statistics were created in order to validate our models.

**Fig. 6:**
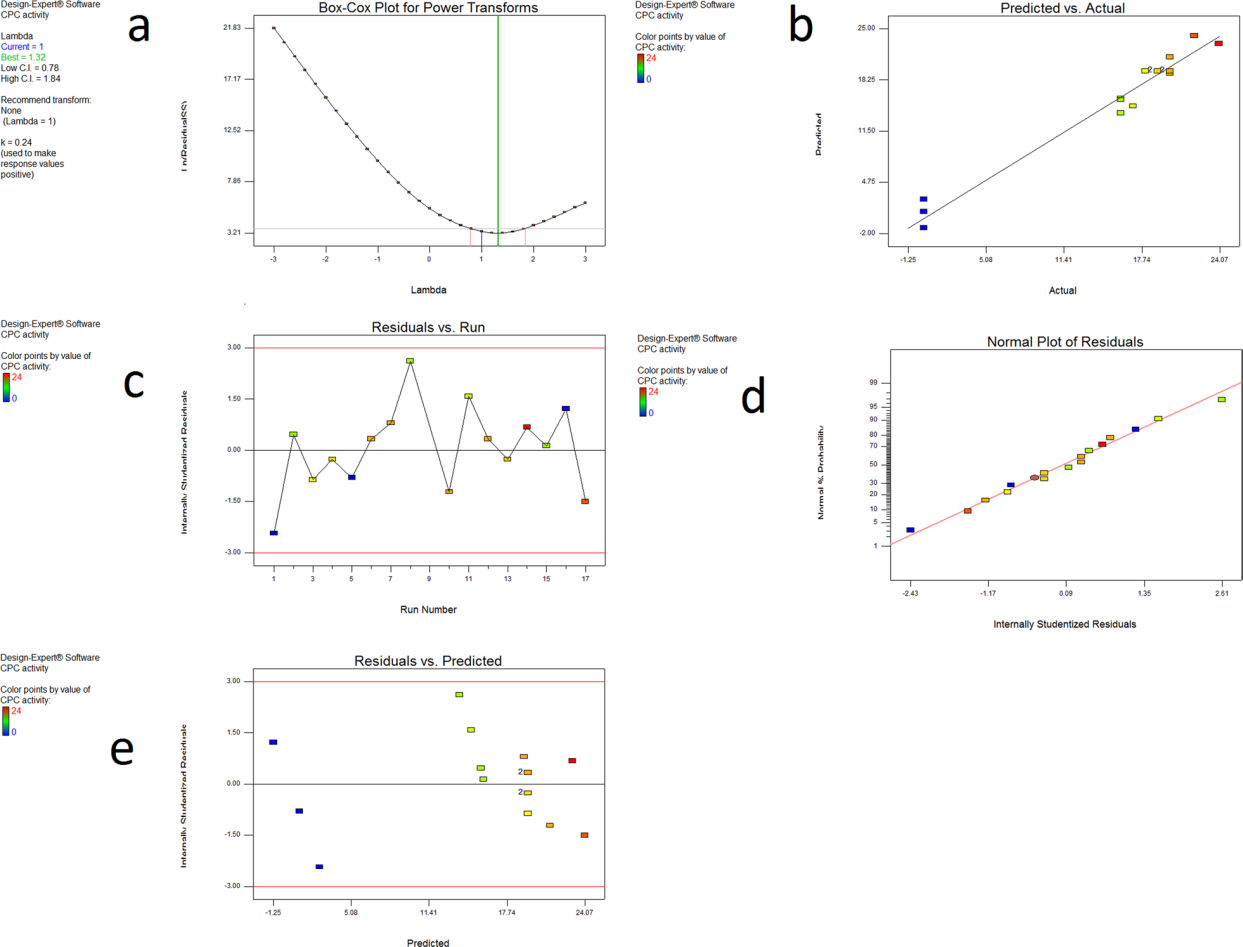
Model Diagnostics **(a)** Box-Cox plot for Power Transforms; **(b)** Predicted vs. actual plot; **(c)** Residuals vs. Run plot; **(d)** Normal plot of Residuals; (**e**) Residuals vs. predicted
- Predicted vs. actual plot
  The plot revealed that the distribution of the points was close to the straight line, indicating that the actual values were extremely similar to those predicted, as seen in Fig. 6b. It helps to detect observations that are not well predicted by the model. Areas of over- or under-prediction are indicated by clusters of points above or below the line.
- Residuals vs. run plot
  It detects any hidden variables that may affect the response throughout the experiment. The plot shows a random scatter. Trends imply the presence of a time-related variable in the background. As seen in Fig. 6c, the points were dispersed about zero, showing that the model fit the data.
- Normal plot of the Residuals
  As can be seen in Fig. 6d, the plot revealed that the points follow a straight line, indicating that the residuals have a normal distribution.
- Residuals vs. predicted
  In this figure, outliers are also investigated at. Runs with residuals outside the plot's red lines are considered outliers. An observation that the model does not adequately fit is known as an outlier. It serves as a visual check for the constant variance supposition. The spots are dispersed at random in Fig. 6e.

- Box Cox plots
  The created model's Box-Cox plots for power transformation revealed that there was no need for additional transformation because the current lambda value (lambda = 1) is within the 95% confidence interval of the best lambda value, as shown in Fig. 6a.

### Experimental confirmation test

**Fig. 7:**
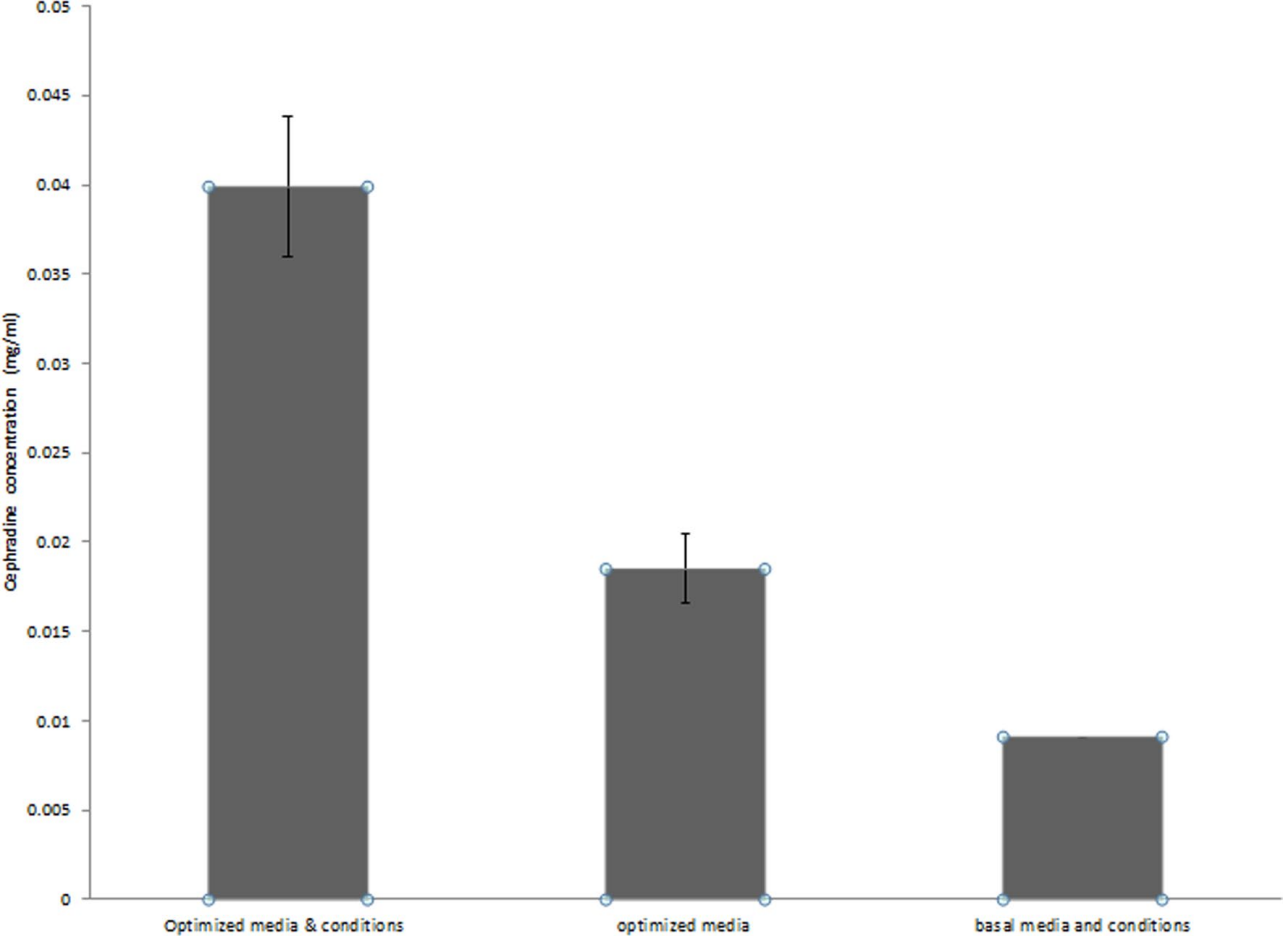
Comparison of CPC activity by *Acremonium chrysogenum* W42-I utilizing optimized and un-optimized conditions

The model's accuracy was confirmed when CPC was produced using the suggested optimal amounts of the three variables (4 incubation days, 1% v/v inoculum, and initial pH 4), in a variant of the basal medium comprising 3 g/L L-Met and 4.7%v/v soybean oil. The resulting inhibition zone of 24.667 mm was extremely close to that predicted by the model (25.6617 mm). This value corresponded to a standard cephradine antibacterial activity of 39.89 µg/mL. The optimal values employed led to increases in CPC production of 2.15 times over those achieved using optimized media but un-optimized culture conditions (18.49 µg/mL) and 4.43 times over those obtained using both un-optimized media and un-optimized culture conditions (9 µg/L) (Fig. 7).

### Effect of gamma irradiation

**Fig. 8:**
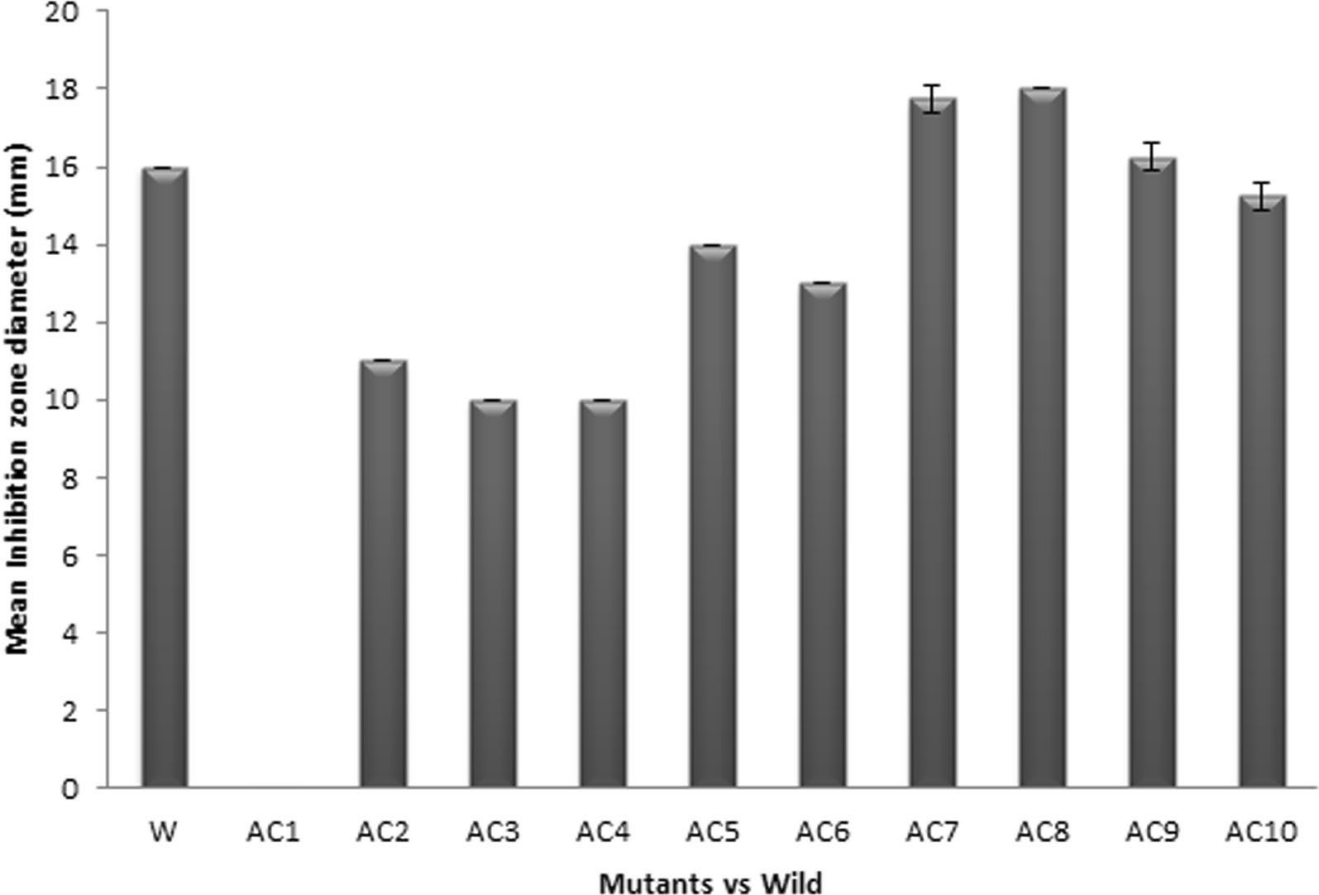
The antibacterial activity of the ten selected Mutants vs. Wild type

It was discovered that 2 KGy is the ideal gamma radiation dose for mutations that result in 99.99% death. On tryptic soy agar plates, gamma irradiation of the wild-type strain resulted in the incidence of ten morphologically altered colonies as shown in Additional file 1: Figs. S2, S3. Figure 8 illustrates the bioassay results for these colonies' antibacterial activity. The colony morphology of the AC8 mutant revealed a change in the color of the aerial mycelia from white to green faster than that of wild type and this morphological variation was accompanied with increase in the antibacterial activity. Additional file 1: Fig. S4 illustrates how this mutant's activity increased approximately 3.46-fold when grown in optimal media and circumstances compared to the wild-type. Additionally, AC8-mutant cells that were cultured repeatedly showed high genetic stability.

## Discussion

The majority of antibiotics now used in clinical practice are cephalosporins (Das et al. 2019). *A. chrysogenum* is the primary strain utilized in the industrial manufacture of cephalosporin C (CPC). The advantages of CPC include its strong antibacterial activity and wide antibacterial range. The yield and titer of cephalosporins obtained from *A. chrysogenum*, which is generated by *Penicillium chrysogenum* as well, are significantly lower than those of penicillin (Liu et al. 2022). The production and price of CPC are crucial for the cephalosporins-antibiotic sector because it is the main source of 7-ACA. The Ministry of Science and Technology of China has named CPC fermentation as one of the main scientific and technological achievements during the past 30 years due to the ongoing demand for strain enhancement for *A. chrysogenum*, a producer of CPC. Due to the drawbacks of conventional methods for *A. chrysogenum* strain improvement and the broad use of molecular biology, genetic engineering has emerged as an effective strategy for modifying the antibiotic-producing strain and developing high-yield mutant strains (Hu and Zhu 2016). The current study attempted to optimize culture medium components and environmental circumstances in order to increase CPC production by the examined isolate. Different L-methionine and soybean oil were evaluated for the main production culture. The parameters chosen for optimization via RSM were the degree of acidity (pH), the size of the bacterial inoculum, along with the incubation period. The software used for this step was Design Expert v. 7.0 as previously reported (Ibrahim et al. 2019; El-Housseiny et al. 2021). Following that, CPC production was increased through mutagenesis utilizing gamma (ɣ) radiation. The efficacy of CPC as an antibacterial agent was bioassessed against the microorganism *Staphylococcus aureus*; strain number ATCC 25923 at the end of each run.

Not only the composition of the culture medium but also the surrounding environment frequently impact the biosynthesis of certain active metabolites by microorganisms (El-Housseiny et al. 2021; Ibrahim et al. 2019). L-methionine and soybean oil are significant components in CPC production media, that act a big role in increasing CPC production. As it supplies sulphur for the synthesis of cephalosporin, L-methionine is a preferred medium ingredient for cephalosporin biosynthesis (Gohar et al. 2013). Besides, L-methionine is the major stimulant to the arthrospore formation that is correlated with CPC production, and is important in induction of four enzymes in CPC biosynthesis (Lotfy 2007; Martín and Demain 2002). Concerning soybean oil, it contains different amino acids from which the CPC biosynthesis process begins (Martin and Demain 1980). Soybean oil supplementation increases CPC production but has little effect on morphology, despite the fact that in batch or fed-batch cultures it occasionally seems like morphology and productivity are connected (Sándor et al. 2001). Consequently, for the main production medium, different L-methionine and soybean oil concentrations were tested as described previously in Additional file 1: Table S1. The optimum media was that which contained 3 g/L L-methionine and 4.7% v/v soybean oil.

The ambient fermentation conditions required to be optimized once the culture media for the manufacture of CPC were optimized. This was achieved using RSM, a helpful mathematical and statistical method for choosing experimental design, showing the relatedness of the components and the ideal integration of parameters, as well as for predicting responses (Ahsan et al. 2017). The Design Expert® v. 7.0 was used for experiment design, mathematical representation, statistical univariate analysis, production of response surfaces, contour, and diagnostic plots.

The 3D response surfaces and respective contour plots provide comprehensive information on the interrelationship between two of the variables, hence allow the prediction of the settings conducive to the highest production (Ghribi et al. 2012). With the help of these plots and a numerical optimization algorithm, the inoculum size of 1% v/v, pH of 4, and incubation period of 4 days were discovered to be the ideal conditions for maximum CPC production. When CPC was produced using the proposed levels, the inhibition zone measured 24.667 mm, which was extremely near to the value estimated by the model (25.6617 mm), demonstrating the validity of the model. This value was calculated using a standard curve using standard cephradine, which has an antibacterial activity of 39.89 µg/mL.

The results were methodically examined by ANOVA, and it proved the adequacy of the model. It also evinced the significant influence the factors possess when producing CPC (Nisha et al. 2023). The P value decides whether the results are significant or not (Lang et al. 1998). The Fisher's F-test demonstrates this with a very low probability value [(P value < 0.000)] (Inamdar et al. 2013). The F value was 48.32, revealing the model’s significance. This F value had 0.01% probability of occurring due to chance. The multivariate models turned out to be significant thus can be used to precisely represent CPC production, according to the ANOVA results for the models. Furthermore, adjusted coefficient of determination (Adj R^2^) results demonstrated a high level of agreement with Pred R2. The difference should be less than 0.2 (Nisha et al. 2023). In our model, R^2^ value of 94.89% (0.9489) showed that the model was highly significant. The CV represents the precision of the treatment comparison. As the CV value increases, the experiment's reliability falls (Ghribi et al. 2012). The low value of the study revealed the great accuracy of the experimental results. Adequate (Adeq.) precision is evaluates the signal-to-noise ratio. An adequate precision ratio of 21.475 was obtained in this model, indicating an appropriate signal. This model is useful for navigating the design space.

The effects of each variable on their own and in combination have an impact on the production of the antibiotic. Tools that make it easier to fully understand the design space influenced by these factors include 3D surface plots and 2D contour plots. As a result, graphical summaries for case statistics were made, to validate the derived models. The Box-Cox plot not only guides the decision regarding which power law transformation to use, but it also suggests whether or not the response should be measured using a different scale. Because the current lambda was within the 95% confidence interval, no transformation was recommended. The Predicted versus Actual plot is applied to compare experimental response values with model predictions to evaluate the results quantitatively. The Residuals versus Run plot displays the residuals in relation to the experimental run order (El-Housseiny et al. 2021). According to the findings of this investigation, these produced graphs demonstrate the models' validity.

According to the ANOVA results, pH and incubation length were also found to have a significant effect on CPC formation. A longer incubation normally improves the organism's development and growth-related activities up to a point, after which there may be a decline in bacterial activity due to nutritional inadequacy (Gohar et al. 2019). In the presented investigation, maximum CPC production was predicted to be obtained after 4 days incubation. The incubation period has a significant impact on *A. chrysogenum*'s ability to produce cephalosporin because it causes the fungal mycelium to differentiate into several morphological forms, which in turn affect the biosynthesis of cephalosporin. Swollen hyphal fragments are the most appropriate morphological form for the synthesis of cephalosporin. Following that, there is a decrease in cephalosporin production as a result of the transformation of inflated hyphal fragment into conidia. Conidia are not related to the production of cephalosporin. (Gohar et al. 2019). Another crucial factor in the expansion of the microorganism and the synthesis of secondary metabolites is the size and age of the inoculum. The performance of the fermentation for the generation of secondary metabolites is significantly influenced by the physiological state of the inoculum when it is transferred to the production stage (Gohar et al. 2019).

Results, using RSM with optimized medium, suggested that the optimum pH is 4. The very brief CPC manufacturing period was caused by the higher pH value attained. Although regulating pH in the shake flask method is difficult, lowering the starting pH is a typical strategy (Cuadra et al. 2008). Given the facts, pH is a crucial factor to consider while studying CPC biosynthesis, as we have seen that the manufacturing of this antibiotic only occurs within a specific pH range (López-Calleja et al. 2012). In the current study, lowering the initial pH was a helpful method that likely enabled for lower pH values to be maintained for a longer period of time throughout culture. This raised CPC yields by 29% and production time from 1 to 2 days. Our optimum pH is similar to that obtained in other statistical study done for Egyptian soil *Acremonium chrysogenum* isolate (Lotfy 2007). It is important to know that the transfer of the results obtained in this study to the industrial production process is to be tested in further investigations. This will definitely be verified during scaling up of CPC production using a pH-controlled fermenter in our future studies.

In strain development, mutation was induced to boost the beneficial microbial secondary metabolite output (Parekh et al. 2000). Mutation and screening for overproducing microbial mutants resulted in not just high production but also inexpensive costs (Adrio and Demain 2006). AT to CG transversions are the most effective techniques to increase yield. Hitherto, no chemical agent is known to be capable of inducing AT to CG transversions (Baltz 2001). AT to CG transversions, on the other hand, were discovered in cells exposed to gamma radiation (Xie et al. 2004). One type of ionizing radiation, gamma rays are thought to be the most energetic radiations. Gamma radiation causes mutations by breaking single or double-strand DNA through deletion or structural abnormality (Huma et al. 2012). It is also employed depending on the biological materials being used and the desired outcome of the work in order to select the best doses. High doses are utilized in sterilization, medium doses in decontamination, and low doses in mutagenesis (Hoe et al. 2016).

For fungi, gamma irradiation is a powerful mutagenic agent (Mutwakil 2011). In our study, *A. chrysogenum* was exposed to gamma rays, which resulted in ten morphologically altered colonies. These colonies were tested for bioactivity against standard *Staphylococcus aureus* ATCC 25923. The effect of gamma irradiation on cell growth was found to be dose-related. The percentage of killing increases as the gamma radiation dose increases. The 99.99% killing was achieved at a dose of 2 KGy. The highest CPC production was found with mutant AC8. When grown in optimal conditions and medium, this mutant demonstrated a 3.46-fold increase in activity when compared to the wild-type. Similar to this, *Aspergillus flavus* and *Aspergillus ochraceus* gamma-irradiated stains at 2 KGy produced two times more mycotoxins than control strains did (Ribeiro et al. 2011). The same findings were observed with *Aspergillus niger*, a powerful producer of numerous crucial industrial enzymes that is genotypically enhanced by gamma radiation exposure. After an 80 Krad dosage, mutants of *Aspergillus niger* demonstrated increased glucose oxidase synthesis (Zia et al. 2012). After being exposed to gamma radiation, *Aspergillus niger,* which produces the enzymes α- and β galactosidases, produced two times as much of these enzymes (Awan et al. 2011). Additionally, a cellulase-producing mutant of *Aspergillus niger* increased the production of carboxymethyl cellulase and filter paper cellulase at a dose of 2 KGy (Mostafa 2014).

Furthermore, our research showed that the mutant AC8 showed genetic stability after being cultured repeatedly. These findings make gamma radiation, in comparison to other mutagens, an efficient method for causing mutation. In conclusion, maximum CPC concentration was obtained using CPC2 medium containing 3 g/L L-methionine and 4.7%v/v soybean oil. In this study, the results showed that using RSM for optimization of CPC production by *Acremonium chrysogenum* W42-I is a competent and useful tool. The use of RSM enabled us to achieve optimal culture conditions with a small number of experimental attempts. The optimum environmental conditions (inoculum size 1% v/v, pH 4 and an incubation period 4 days), resulted in about 4.43-fold rise in CPC production reaching 39.89 µg/mL. The final model accurately predicted data points that matched the experimental values. These results demonstrated that RSM and experimental design are effective strategies for enhancing environmental variables that increase the production of CPC by the studied isolate.

Mutagenesis caused by gamma irradiation could result in production-related morphological alterations in *A. chrysogenum* W42-I. Following re-plating of the mutants, the same morphological modifications were observed, and the CPC production of mutant AC8 remained constant after each culture. When grown in optimal conditions and medium, an AC8 mutant demonstrated a 3.46-fold increase in activity when compared to the wild-type. As a result, the *A. chrysogenum* mutant AC8 is a promising industrial strain for the synthesis of CPC.

### Supplementary Information

**Additional file 1: Table S1.** Different concentrations of L-methionine and soybean oil used in CPC production media optimization. (L-methionine was tested by constant 4.7% v/v soybean oil and soybean oil was tested by constant 3 g/l L-methionine). **Table S2.** The RSM runs for three levels of the three selected environmental factors. **Table S3.** Analysis of variance (ANOVA) for Response Surface Reduced Quadratic Model. **Figure S1.** HPLC Chromatograms of: A: Standard CPC; B: Chromatogram of the CPC produced by *A. chrysogenum* W42-I. **Figure S2.** Photographs of mutants’ colonies resulted after exposure of *Acremonium chrysogenum* to gamma rays. **Figure S3.** Photographs of AC8-mutant (a) and *Acremonium chrysogenum* wild type (b) colonies. **Figure S4.** The promising increase in the antibacterial activity of Mutant AC8 when cultured in the optimal medium.

## Data Availability

All data generated or analyzed during this study are included in this published article and Additional file 1.
