# Supplementary material for: Statistical optimization and gamma irradiation on cephalosporin C production by Acremonium chrysogenum W42-I
Source: AMB Express. 2023 Dec 11;13:142. doi: 10.1186/s13568-023-01645-5 (PMC10713967; doi:10.1186/s13568-023-01645-5)
Supplement: Supplementary file 1 — Additional file 1: Table S1. Different concentrations of L-methionine and soybean oil used in CPC production media optimization. (L-methionine was tested by constant 4.7% v/v soybean oil and soybean oil was tested by constant 3 g/l L-methionine). Table S2. The RSM runs for three levels of the three selected environmental factors. Table S3. Analysis of variance (ANOVA) for Response Surface Reduced Quadratic Model. Figure S1. HPLC Chromatograms of: A: Standard CPC; B: Chromatogram of the CPC produced by A. chrysogenum W42-I. Figure S2. Photographs of mutants’ colonies resulted after exposure of Acremonium chrysogenum to gamma rays. Figure S3. Photographs of AC8-mutant (a) and Acremonium chrysogenum wild type (b) colonies. Figure S4. The promising increase in the antibacterial activity of Mutant AC8 when cultured in the optimal medium. [file 13568_2023_1645_MOESM1_ESM.docx]

**-Journal name**: AMB Express

**-Manuscript Title**: Statistical optimization and Gamma irradiation on Cephalosporin C production by *Acremonium chrysogenum* W42-I

Asmaa A. Ibrahim^1^, Ghadir S. El-Housseiny^1^, Khaled M. Aboshanab*^1^, Ansgar Startmann^2^_,_ Mahmoud A. Yassien^1^, Nadia A. Hassouna^1^,

^1^Department of Microbiology and Immunology, Faculty of Pharmacy, Ain shams University, Organization of African Unity St., POB: 11566, Abbassia, Cairo, Egypt

^2^ W42 Industrial Biotechnology GmbH, 44227 Dortmund, Germany.

* Corresponding Author: Prof. Dr Khaled Aboshanab, PhD

Address: Department of Microbiology and Immunology, Faculty of Pharmacy, Ain Shams University, Organization of African Unity St., POB: 11566, Abbassia, Cairo, Egypt.

E-mail:[aboshanab2012@pharma.asu.edu.eg](mailto:aboshanab2012@pharma.asu.edu.eg)

ORCID: <https://orchid.org/0000-0002-7608-850X>

Tel: (202)28434595

Mobile: 01007582620

Fax: (202)24051107

E-mails of Coauthors:

Asmaa A. Ibrahim: [asmaa.zaki@pharma.asu.edu.eg](mailto:asmaa.zaki@pharma.asu.edu.eg)

Ghadir S. El-Housseiny: [ghadir.elhossaieny@pharma.asu.edu.eg](mailto:ghadir.elhossaieny@pharma.asu.edu.eg)

Ansgar Stratmann: [a.stratmann@w42biotechnology.de](mailto:a.stratmann@w42biotechnology.de)

Mahmoud A. Yassien: [mahmoud.yassien@pharma.asu.edu.eg](mailto:mahmoud.yassien@pharma.asu.edu.eg)

Nadia A. Hassouna: [nadia.hassouna@pharma.asu.edu.eg](mailto:nadia.hassouna@pharma.asu.edu.eg)

**Table S1** Different concentrations of L-methionine and soybean oil used in CPC production media optimization. (L-methionine was tested by constant 4.7% v/v soybean oil and soybean oil was tested by constant 3 g/l L-methionine).

| L-methionine (g/L) | Soybean oil (% v/v) |
| --- | --- |
| 0.5 | 1 |
| 1 | 2 |
| 2 | 3 |
| 3 | 4 |
| 4 | 4.7 |
| 5 | 6 |
| 6 | 7 |

**Table S2** The RSM runs for three levels of the three selected environmental factors

| Run Order | pH (A) | Inoculum size (B, % v/v) | Incubation period (C, days) |
| --- | --- | --- | --- |
| 1 | 6.5 | 1 | 1 |
| 2 | 9 | 2.5 | 1 |
| 3 | 6.5 | 2.5 | 3.5 |
| 4 | 6.5 | 2.5 | 3.5 |
| 5 | 9 | 2.5 | 6 |
| 6 | 6.5 | 2.5 | 3.5 |
| 7 | 4 | 2.5 | 6 |
| 8 | 4 | 2.5 | 1 |
| 9 | 6.5 | 4 | 1 |
| 10 | 6.5 | 1 | 6 |
| 11 | 9 | 1 | 3.5 |
| 12 | 6.5 | 2.5 | 3.5 |
| 13 | 6.5 | 2.5 | 3.5 |
| 14 | 4 | 1 | 3.5 |
| 15 | 9 | 4 | 3.5 |
| 16 | 6.5 | 4 | 6 |
| 17 | 4 | 4 | 3.5 |
| Factor | Name | Level (-1) | level (+1) |
| A | pH | 4 | 9 |
| B | Inoculum size (% v/v) | 1 | 4 |
| C | Incubation period (days) | 1 | 6 |

**Table S3** Analysis of variance (ANOVA) for Response Surface Reduced Quadratic Model

| **Source** | **Sum of Squares** | **Df** | **Mean Square** | **F Value** | **p- value** |
| --- | --- | --- | --- | --- | --- |
| Model | 921.34 | 6 | 153.56 | 48.33 | < 0.0001 |
| A-ph | 136.13 | 1 | 136.13 | 42.84 | 0.0001 |
| B-inoculum size | 1.50 | 1 | 1.50 | 0.47 | 0.5093 |
| C-incubation period | 33.84 | 1 | 33.84 | 10.65 | 0.0098 |
| AC | 100.00 | 1 | 100.00 | 31.47 | 0.0003 |
| BC | 331.35 | 1 | 331.35 | 104.28 | < 0.0001 |
| C^2^ | 179.92 | 1 | 179.92 | 56.62 | < 0.0001 |
| Residual | 28.60 | 9 | 3.18 |  |  |
| Pure Error | 2.80 | 4 | 0.70 |  |  |
| Cor Total | 949.94 | 15 |  |  |  |


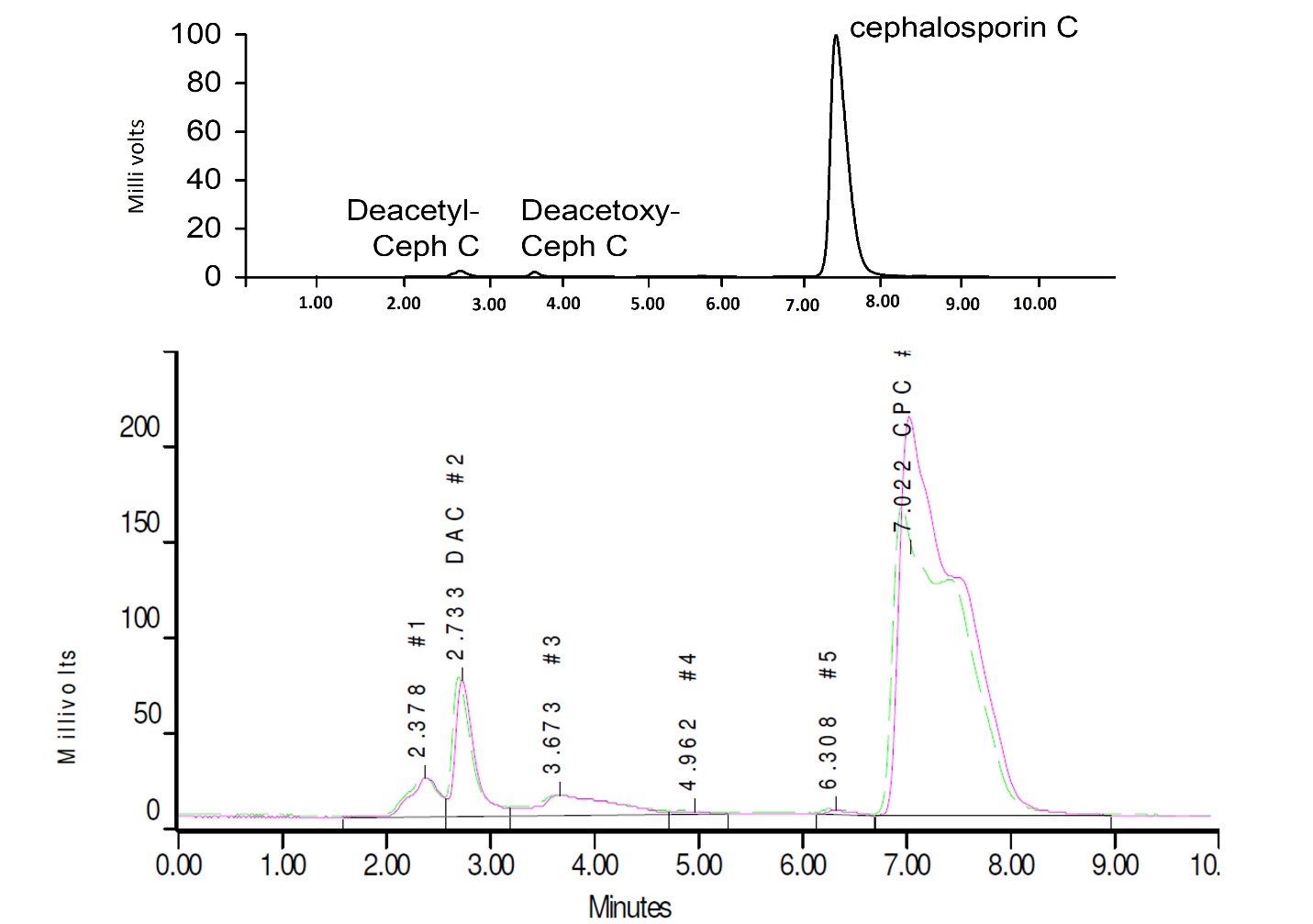


A

B

**Figure S1.** HPLC Chromatograms of: A: Standard CPC; B: Chromatogram of the CPC produced by *A. chrysogenum* W42-I.


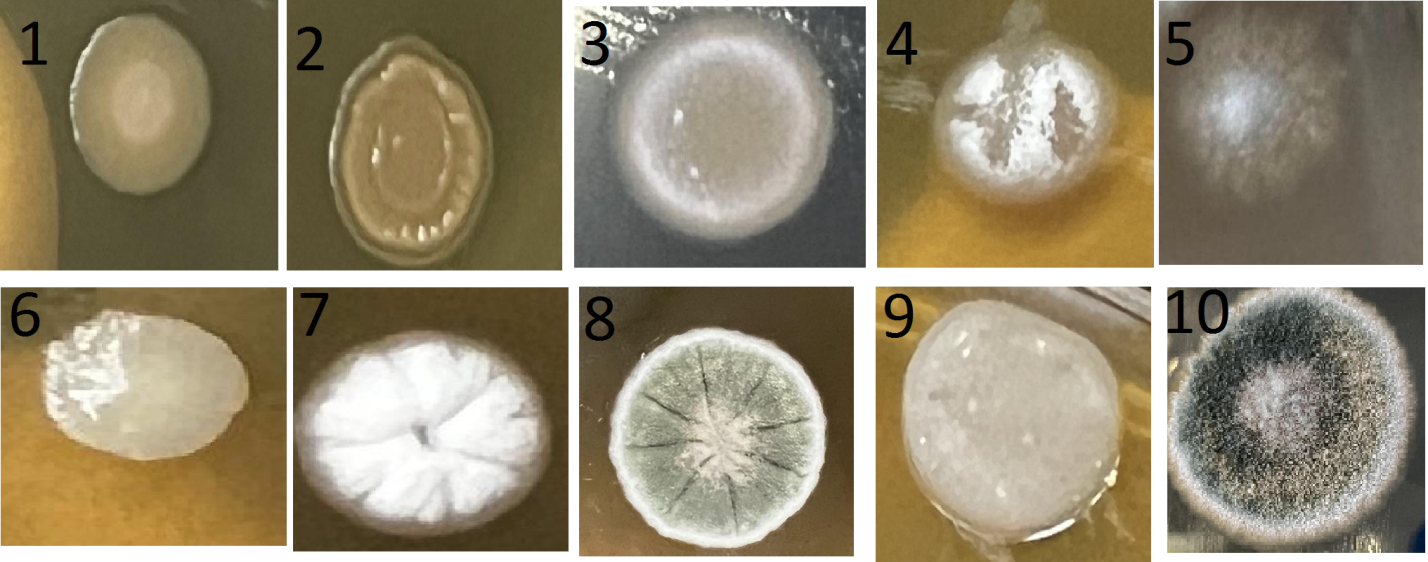


**Figure S2** Photographs of mutants’ colonies resulted after exposure of *Acremonium chrysogenum* to gamma rays


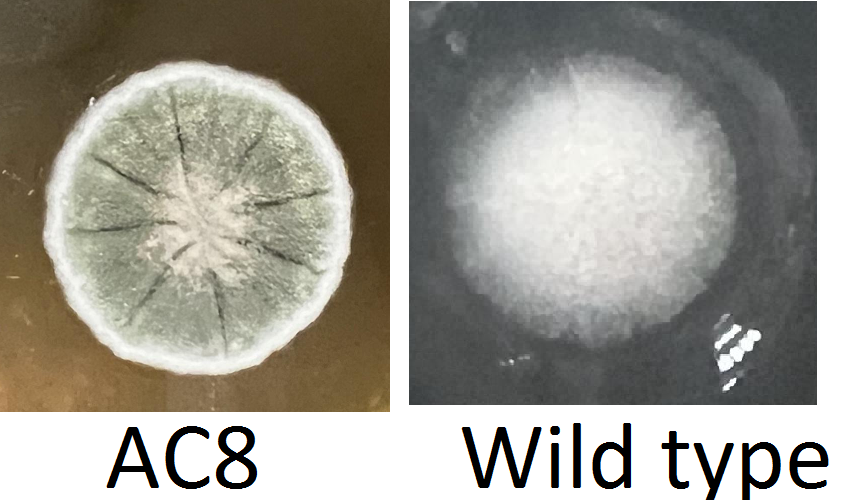


b

a

**Figure S3** Photographs of AC8-mutant (a) and *Acremonium chrysogenum* wild type (b) colonies


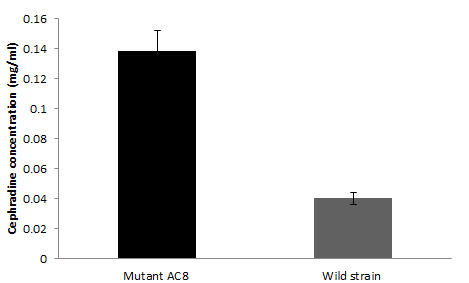


**Fig. S4** The promising increase in the antibacterial activity of Mutant AC8 when cultured in the optimal medium
